# Association of decreased C1q/tumor necrosis factorrelated protein-5 levels with metabolic and hormonal disturbance in polycystic ovary syndrome

**DOI:** 10.4274/jtgga.galenos.2018.2018.0027

**Published:** 2019-05-28

**Authors:** Mehmet Çalan, Murat Alan, Pınar Alarslan, Gökçen Ünal Kocabaş, Giray Bozkaya, Ahmet Çağdaş Acara, Behnaz Aslanipour, Özge Fenercioğlu, Ahmet Murat Işıl, Aslı Güler

**Affiliations:** 1 Clinic of Internal Medicine, Division of Endocrinology and Metabolism, University of Health Sciences, İzmir Bozyaka Training and Research Hospital, İzmir, Turkey; 2 Clinic of Obstetrics and Gynecology, University of Health Sciences, İzmir Tepecik Training and Research Hospital, İzmir, Turkey; 3 Clinic of Clinical Biochemistry, University of Health Sciences, İzmir Bozyaka Training and Research Hospital, İzmir, Turkey; 4 Clinic of Emergency Medicine, University of Health Sciences, İzmir Bozyaka Training and Research Hospital, İzmir, Turkey; 5 Department of Biotechnology, Ege University Faculty of Medicine, Graduate School of Natural and Applied Sciences, İzmir, Turkey; 6 Clinic of Family Physician, University of Health Sciences, İzmir Bozyaka Training and Research Hospital, İzmir, Turkey

**Keywords:** Polycystic ovary syndrome, C1q/tumor necrosis factor-related protein-5, insulin resistance, body mass index, free-androgen index

## Abstract

**Objective:**

C1q/tumor necrosis factor-related protein-5 (CTRP5) is a novel peptide hormone involved in the metabolism of energy regulation. Polycystic ovary syndrome (PCOS), which is a reproductive and metabolic disorder, is associated with insulin resistance. The aim of the current study was to compare circulating levels of CTRP5 in women with and without PCOS and to investigate possible associations between CTRP5 and metabolic-hormonal parameters.

**Material and Methods:**

The present cross-sectional study contained 80 women with PCOS and 80 age and body mass index-matched women without PCOS. Circulating levels of CTRP5 were calculated using an enzyme-linked immunosorbent assay. We also measured hormonal and metabolic parameters.

**Results:**

Patients with PCOS had lower levels of circulating CTRP5 compared with women without PCOS (6.90±2.64 vs 11.73±3.66 ng/mL, p<0.001). CTRP5 was negatively correlated with insulin resistance, free-androgen index, and body mass index in both the PCOS and control groups. Moreover, patients with PCOS who had insulin resistance showed lower circulating CTRP5 levels compared with those without insulin resistance. In both the control and PCOS groups, overweight subjects had lower circulating levels of CTRP5 compared with participants of normal weight. Logistic regression analyses indicated that subjects in the lowest tertile for CTRP5 level had higher risk for PCOS compared with those in the highest tertile of CTRP5.

**Conclusion:**

Decreased circulating levels of CTRP5 were associated with higher risk of PCOS, as well as having metabolic disturbance among women with PCOS.

## Introduction

Polycystic ovary syndrome (PCOS) is known as a common metabolic and reproductive disease in women of reproductive age, which is characterized by ovulatory dysfunction, clinical and/or laboratory hyperandrogenism, and polycystic ovaries. Despite a lack of clear information about the pathophysiology of PCOS, genetic and environmental factors are believed to be influential in the development of the disease (1,2,3). Insulin resistance, glucose and lipid metabolism dysfunction, and obesity are frequently reported in women with PCOS. PCOS is also associated with low-grade chronic inflammation. Moreover, both insulin resistance and low-grade inflammation induce hormonal and metabolic abnormalities in women with PCOS (1,4). Changes in expression levels of various peptides in adipose tissue such as adiponectin in women with PCOS lead to hormonal and metabolic dysfunctions (5,6). 

C1q/tumor necrosis factor-related protein-5 (CTRP5), a secreted peptide hormone and adiponectin paralog, is involved in energy metabolism, including glucose and lipid metabolism. CTRP5, which is expressed in many tissues such as adipose, myocyte, and liver (7,8,9,10), has the ability to induce phosphorylation of AMP-activated protein kinase (AMPK), thus stimulating glucose uptake and fatty acid oxidation (8,11). Hence, CTRP5 is highly expressed in obese and diabetic animal subjects (12). It has been illustrated that obesity level in humans is proportionate to enhanced expression in adipose tissue (13). On the other hand, genetically CTRP5-deficient mice showed improved insulin action (12). In accordance with these results, patients with type 2 diabetes (T2DM) were revealed to have lower CTRP5 levels, and CTRP5 was negatively correlated with both body mass index (BMI), as well as insulin resistance (14). It has also been reported that CTRP5 induces inflammation and proliferation in human aortic smooth muscle cells by activating a variety of pathways (15). In addition, a positive association was observed between CTRP5 and C-reactive protein (CRP) in subjects with chronic obstructive pulmonary disease (16).

In the current study, we compared CTRP5 levels in women with PCOS and control participants without PCOS and investigated relationships between CTRP5 and hormonal or metabolic parameters.

## Material and Methods

### Ethics

Ethical approval was issued by the ethics committee of İzmir Bozyaka Training and Research Hospital for the present study (no: 2. GOA/2016) and all participants provided written informed consent. The study was conducted and accomplished according to the Declaration of Helsinki (2008). 

### Study design and participants

This case-control study included two groups: 80 subjects with PCOS and a group of 80 age and BMI-matched women with normal menstruation. Participants aged 18-45 years were recruited. The study was conducted between June 2016 and January 2017 in the Endocrinology Department of the Bozyaka Training and Research Hospital in İzmir, Turkey. We consecutively recruited subjects who met all of the exclusion and inclusion criteria of the study to reach to the planned population. All participants had a BMI >18.5 kg/m^2^ and ≤35 kg/m^2^, and none had alcohol and tobacco addiction. The same researcher performed all examinations, and obtained detailed histories. All participants were subjected to 2-h 75-g oral glucose tolerance standard test (OGTT). All participants were drug naive.

### PCOS group

The patients in the PCOS group were selected based on the Rotterdam Consensus Criteria (2003) and other possible causes of hyperandrogenism and ovulatory dysfunction were also excluded from the study (17). The presence of at least two of three criteria is needed to diagnose of PCOS, but we selected women who met all three criteria of Rotterdam Consensus Criteria to achieve homogeneity among the subjects. These criteria include 1- anovulation or oligoovulation; 2- either biochemical or clinical symptoms of hyperandrogenism, using Ferriman-Gallwey (FG) score to assess hirsutism (18); and 3- the existence of ≥12 follicles measuring from 2 to 9 mm in diameter or ovarian volume >10 mL (without a cyst or dominant follicle in one of the ovaries). Ultrasonographic signs consistent with PCOS in a single ovary were sufficient for diagnosis (M.A.). 

Patients with FG score ≥8 were considered hirsute. Biochemical markers of hyperandrogenism were defined if serum levels of testosterone (normal range: 0.52 to 2.42 nmol/L), and/or dehydroepiandrosterone sulfate (DHEA-S) (normal range: 10-248 μg/dL), and/or free androgen index (FAI) ≥5% (19) were higher than the maximum limit of reference intervals.

### Control group

The control group of comprised women who had visited the endocrinology and/or gynecology clinics for checkup and volunteer employees from the hospital. All controls had normal menstrual cycles. Disorders such as problems in concomitant health issue, acne, hyperandrogenism, or hirsutism symptoms were not detected in any patients in the control group. 

### Exclusion criteria

Participants who were pregnant/breastfeeding or had any other causes or signs of menstrual irregularity and/or androgen excess, such as adrenal, pituitary, or thyroid disorders (including congenital adrenal hyperplasia, hyperprolactinemia, Cushing’s syndrome, hyperprolactinemia and galactorrhea) were not included. Other exclusion criteria included reduced glucose tolerance, type 1/2 diabetes, or history of gestational diabetes; history of hypertension, hyperlipidemia, liver/renal disorders, coronary artery disease, congestive heart failure, malignancy or acute infection (in the past two weeks); and chronic inflammatory or autoimmune disorders. Furthermore, participants with hormonal contraception and/or anti-androgen use in the past six months and those taking medications for treatment of hypertension, insulin resistance, hyperglycemia, dyslipidemia and obesity were excluded. 

### Anthropometric evaluation

Anthropometric measurements including age, waist circumference (cm), both weight (kg) and height (cm) were analyzed when the participants were wearing casual clothes and barefoot. The distance between the lower rib margin and the iliac crest at the end of a gentle expiration was used to find the waist circumference. Blood pressure was measured with the participants in a resting position after a 15 minute resting period. BMI was calculated considering the formula of weight (kg)/ height in meters squared (m^2^).

### Biochemical evaluation

Venous blood samples were obtained from all participants in the early follicular phase (day 3 to 5) of spontaneous or progesterone-induced menses, in the morning (between 08:00-09:00) after at least a 10 hour fast. The blood samples were held at room temperature for at least 30 minutes to allow coagulation. The samples were then centrifuged at 2000×g for 15 minutes and serum aliquots were maintained at -80 °C until analysis of CTRP5. Fasting blood glucose (FBG), glycated hemoglobin A1_C_ (HbA1_C_), serum insulin, high-density lipoprotein cholesterol (HDL-C), total amount of triglyceride, cholesterol and testosterone, DHEA-S, luteinizing hormone (LH), sex hormone of binding-globulin (SHBG), follicle-stimulating hormone (FSH), estradiol (E_2_), 2-h plasma glucose following 75-g OGTT (2-h OGTT) and high-sensitivity of CRP levels were also measured. The levels of low-density lipoprotein cholesterol (LDL-C) were calculated considering the following formula: LDL-C=total cholesterol - (HDL-C + triglycerides/5). FBG, 2-h OGTT and hs-CRP of serum, serum, total cholesterol, triglycerides, and HDL-C were measured considering an auto-analyzer (Olympus AU 2700 Beckman Coulter Inc, CA, USA) with dedicated kits (Beckman Coulter Inc, CA, USA). The levels of insulin in serum were measured by means of chemiluminescent microparticle immunoassay (CMIA) with dedicated kits (Beckman Coulter Inc, CA, USA) along with auto-analyzer (UniCel DxI 800, Beckman Coulter Inc, CA, USA). High-performance liquid chromatography (Variant II Turbo, Bio-Rad, CA, USA) was used to measure HbA1_C_ levels. LH, FSH, E_2_, DHEA-S, the levels of total testosterone and SHBG were also measured using CMIA (UniCel DXI 800, Beckman Coulter Inc., CA, USA). We calculated FAI by the following formula as (total testosteron/SHBG)×100. We used the homeostasis model assessment of insulin resistance (HOMA-IR) for the calculation of insulin resistance: fasting insulin (µU/mL)×fasting glucose (mg/dL)/405 (20).

### Measurement of circulating CTRP5 by ELISA

Commercially available human ELISA kits (E-EL-H4186, Elabscience-Biotech Co. Ltd, Wuhan, China) were used to measure serum CTRP5 levels (in duplicate) in accordance with the manufacturer’s instructions. The intra-assay coefficient of variability (CV) showed a rate <6% and inter-assay CV showed a rate <8%. The detectable range for serum CTRP5 was 0.31 to 20 ng/mL.

### Statistical analysis

### Power analysis

The minimum number of participants required for a study power of 0.90 and α=0.05 was determined using G Power 3.0.10 G software for Windows (Heinrich-Heine-Universität Düsseldorf, Düsseldorf, Germany) (21), based on the results of our pilot study on circulating CTRP5 levels. We evaluated 25 women with PCOS (CTRP5 levels: 5.38±3.13 ng/mL) and 25 women as controls (CTRP5 levels: 8.20±3.98 ng/mL). According to this analysis, a minimum of 68 subjects were needed in each group. The investigation of all data analyses was completed using the Statistical Package for the Social Sciences software version 18.0 (SPSS Inc. Chicago, IL, USA). Kolmogorov-Smirnov test showed that the numeric variables conformed to normal distribution. The data are stated as mean ± standard deviation. A t-test was considered for the comparison of laboratory and demographic characteristics between the two groups. The PCOS group was separated into two different subgroups: patients with insulin resistance (HOMA-IR >2.71) and those subjects without insulin resistance (HOMA-IR ≤2.71) (22). CTRP5 levels in the PCOS subgroups were also compared using the t-test. Relationships between CTRP5 and other variables were assessed using Pearson’s correlation coefficient, and a linear regression model was formed to assess the presence of independent associations between CTRP5 and metabolic-hormonal parameters including FAI, BMI, and HOMA-IR. The variance inflation factor (VIF) of independent variables was calculated to determine multicollinearity. Variables with VIF >2.5, such as FBG and waist circumference, were not used in the model. We also adjusted the model for some parameters such as age, hs-CRP, and lipid parameters, as well as PCOS status. To estimate the possible association between CTRP5 levels (in tertiles) and PCOS risk, odds ratios (OR) were calculated using multivariate logistic regression analysis. Possible confounders such as BMI, age, HOMA-IR, FAI, and lipid parameters were included in the model for adjustment. The compatibility of the model was evaluated using the Hosmer-Lemeshow test (p>0.05). Confidence intervals (CI) were calculated at 95% and two-sided p values <0.05 were accepted as statistically significant. 

## Results

### Laboratory and clinical characteristics of the population of the study

The comparison of the clinical and laboratory parameters of the enrolled groups are presented in Table 1.

**Figure 1:**
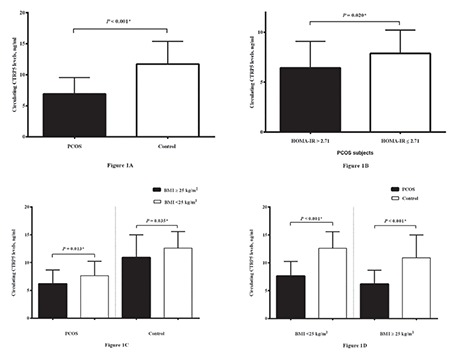
a) Circulating levels of CTRP5 in both control and PCOS subjects. b) Circulating levels of CTRP5 in PCOS patients having insulin resistance (HOMA-IR >2.71) and PCOS subjects with normal insulin levels (HOMA-IR ≤2.71). c) Circulating CTRP5 levels in both overweight/obese (in which BMI ≥25 kg/m^2^) and normal weight (in which BMI <25 kg/m^2^) participants. d) Circulating CTRP5 levels in PCOS and control groups based on BMI status *Statistically significant; CTRP5: C1q/tumor necrosis factorrelated protein-5; PCOS: Polycystic ovary syndrome; HOMA-IR: Homeostasis model assessment of insulin resistance; BMI: Body mass index

CTRP5 levels were notably lower in women with PCOS than in those without PCOS (6.90±2.64 vs 11.73±3.66 ng/mL, p<0.001) (Figure 1a). The levels of FBG, HOMA-IR and serum insulin were meaningfully higher in patients with PCOS as compared with controls. Moreover, patients with PCOS had markedly higher circulating levels of FAI, total testosterone, hs-CRP, and DHEA-SO_4_ as compared with the controls. In the comparison of CTRP5 levels in the PCOS subgroups with insulin resistance (54 of 80 subjects with PCOS had insulin resistance) and without insulin resistance, CTRP5 level was remarkably lower in among the patients with PCOS with insulin resistance (6.43±2.67 vs 7.88±2.34 ng/mL, p=0.020*) (Figure 1b).

In addition, the study participants were also divided into four groups based on BMI (<25 kg/m^2^ and ≥25 kg/m^2^) and PCOS status. Circulating CTRP5 levels were compared between overweight and normal weight subgroups in both PCOS and control groups using t-tests. The subdivision of the PCOS and control groups based on their BMI showed that 40 participants in the PCOS group and 41 in the control group were overweight (p=0.999). In both groups, the mean values for circulating CTRP5 were meaningfully lower in overweight subjects compared with subjects with normal BMI (PCOS group: 6.17±2.50 vs 7.63±2.61 ng/mL, p=0.013*; control group: 10.89±4.10 vs 12.60±2.95 ng/mL, p=0.035*) (Figure 1c). Moreover, we compared circulating CTRP5 levels in the PCOS and control groups according to their BMI status (Figure 1d). CTRP5 levels were found to be decreased in PCOS group compared with the control group in both overweight and normal weight subjects (p<0.001*).

### Correlation of CTRP5 with other parameters

Pearson’s correlation analysis was used to determine whether CTRP5 was associated with demographic or metabolic-hormonal parameters in the PCOS and control groups, as shown in Table 2. 

We demonstrated that CTRP5 levels were in negatively correlated with waist circumference, BMI, HOMA-IR, FAI, and triglycerides, whereas HDL-C was positively correlated with CTRP5. CTRP5 showed no correlation with blood pressure, FSH, LH, and DHEA-S.

### Multivariate regression analysis

Linear regression analysis was focused to assess the existence of independent relationships between CTRP5 and HOMA-IR, BMI, and FAI. PCOS status, age, hs-CRP, and lipid parameters were included in the regression model to adjust for their potentially confounding effects (Table 3). According to the results of the regression analysis, CTRP5 could show an independently negative association with HOMA-IR, FAI, and BMI.

### Multivariate binary logistic regression analysis

**Figure 2:**
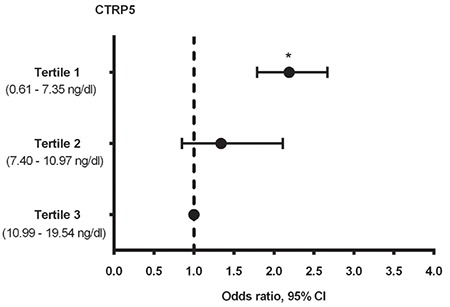
CTRP5 association with PCOS in previously adjusted models as multivariate adjusted OR for PCOS based on CTRP5 tertile (in reference to highest tertile). The model was basically adjusted for HOMA-IR, BMI, age, FAI, and lipid profiles *Statistically significant; CI: Confidence interval; OR: Odds ratio; CTRP5: C1q/tumor necrosis factor-related protein-5; PCOS: Polycystic ovary syndrome; HOMA-IR: Homeostasis model assessment of insulin resistance; BMI: Body mass index; FAI: Free androgen index

Binary logistic regression analysis was conducted to show the probable association between CTRP5 levels (tertile) and the risk of developing PCOS. Potential confounders such as age, HOMA-IR, BMI, FAI, and lipid parameters were included in the model for adjustment (Figure 2). The final results of the abovementioned analysis showed that the subjects in the lowest tertile for CTRP5 displayed meaningfully higher odds of having PCOS risk with respect to the subjects in the highest tertile for CTRP5 [OR=2.19, 95% CI: (1.79-2.67); p=0.021]. There was no remarkable difference in PCOS risk between participants in the second and highest CTRP5 tertiles [OR=1.34, 95% CI: (0.85-2.11); p=0.136].

## Discussion

Patients with PCOS tend to have glucose and lipid metabolism disturbances. It is implicated that some adipokines also result in various metabolic abnormalities in women with PCOS. CTRP5 is a newly defined adipokine, which is involved in energy metabolism; therefore, we tried to evaluate CTRP5 levels in women with PCOS. We found that CTRP5 levels were significantly lower in subjects with PCOS than in controls. Decreased CTRP5 levels were also negatively associated with metabolic and hormonal disturbances. 

Insulin resistance occurs in most patients with PCOS. It is implicated that insulin resistance has a critical role in the pathogenesis of PCOS although the its underlying molecular mechanism in women with PCOS is not fully understood. It is thought to be primarily related to defective insulin-dependent glucose transport into cells. Adipose tissue dysfunction may contribute to the development of insulin resistance in PCOS (1,2,3,4,23). Adipose tissue secretes numerous adipokines involved in regulating metabolic processes; therefore, altered adipose tissue secretion is implicated as one of the main causes of metabolic disorders in patients with PCOS (24,25). Adiponectin, a secreted adipokine, has an essential role in glucose metabolism. Decreased levels of adiponectin also contribute to the development of metabolic disturbance and insulin resistance (26). CTRP5 is a novel peptide hormone with similar structural properties to adiponectin, and it is also involved in energy metabolism (7,8,9,10,11). The structural similarities of CTRP5 to adiponectin revealed a way to clarify the relationship between CTRP5 and metabolic disorders for instance obesity as well as T2DM. The present study is the first to evaluate whether levels of CTRP are altered in women with PCOS compared with controls. We also investigated the link between metabolic/hormonal parameters and CTRP5 in women with PCOS. We found that CTRP5 levels were lower in subjects with PCOS than in controls. We also observed that CTRP5 showed a negative association with insulin resistance markers, BMI and FAI. Linear regression analysis confirmed the negatively independent associations with BMI, insulin resistance, and FAI. Furthermore, we determined that reduced levels of CTRP5 were associated with a high level in risk of having PCOS. Participants in the lowest tertile for CTRP5 had nearly 2.19 times higher risk of having PCOS as compared with those in the highest tertile for CTRP5.

CTRP5 is a member of the CTRP family of novel secreted hormones. Growing evidence supports the presence of a possible link between CTRP5 and metabolic disorders (7,8,9,10,11). Studies investigating the importance of CTRP5 in obesity and diabetes mellitus indicated that increased adipose tissue was associated with elevated CTRP5 secretion (12,13). Contrary to these findings, we detected a highly negative correlation between body weight and CTRP5 levels in the current study. Our findings are consistent with a clinical study in which CTRP5 levels were negatively correlated with BMI (14). There is no certain explanation for these discrepancies. Larger studies are needed to clarify this relationship. 

There are also contradictory data in the literature concerning the effects of CTRP5 on insulin resistance. In one study, it was reported that CTRP5-deficient mice showed reduced hepatic steatosis and improvement of insulin resistance, and treatment with recombinant CTRP5 inhibited insulin-stimulated Akt phosphorylation. Therefore, the authors concluded that CTRP5 was a potential negative controller of the metabolisms of both glucose and insulin sensitivity (12). Interestingly, they reported that nutrition affected the expression of CTRP5, as refeeding resulted in a decrease of CTRP5 expression. In contrast to these findings, Yang and Lee (11) suggested that CTRP5 improved insulin resistance in myocytes. Their results corroborated those of a clinical study in which circulating CTRP5 levels were notably raised in healthy subjects compared with patients with T2DM and non-alcoholic fatty liver disease (NAFLD). In the same study, negative correlations were reported between CTRP5 and insulin, FBG, and insulin resistance, and lower circulating CTRP5 level was determined to be a statistically noteworthy risky factor for T2DM and NAFLD after adjustment for potential confounders (14). In the present study, CTRP5 levels showed a weak and negative correlation with insulin, FBG, and insulin resistance. We also determined that participants in the lowest CTRP5 tertile had higher PCOS risk compared with those in the highest CTRP5 tertile.

There are few reports regarding CTRP5 and lipid metabolism (8,14). CTRP5 was found to be increased in fatty acid oxidation via phosphorylation of AMPK (8). A negative correlation was reported between CTRP5 and triglycerides in a clinical study (14). In the current study, we also reported a weak and negative correlation between CTRP5 and triglycerides, whereas CTRP5 was positively correlated with HDL cholesterol.

PCOS is also known as an inflammatory-based metabolic disorder in which a variety of inflammatory markers are elevated. In our study, we explored why levels of hs-CRP were higher in women with PCOS. It has been suggested that CTRP induces inflammation in vascular smooth muscle cells (15). Moreover, CTRP5 was positively correlated with CRP in subjects with chronic obstructive pulmonary disease (16). However, we detected no notable correlation between CTRP5 and hs-CRP in the present study.

The current study has some limitations. The lack of assessment of other adipokines such as adiponectin is a restriction of this study. The cross-sectional design of the study is another limitation but we realized that cross-sectional studies cannot establish causality; however, they can advance our understanding of the connections between molecules and disorders. 

To sum up, our results designate that decreased CTRP5 levels are linked both with PCOS and metabolic disturbances in this disorder. Reduced CTRP5 may be among the primary activators of PCOS or could simply be a result of the metabolic and hormonal changes that occur in PCOS. To clarify this point, more basic research is desired to explicate the role of CTRP5 in detail. Understanding the role of CTRP5 in hormonal and metabolic processes may lead to new treatments of metabolic abnormalities, as well as PCOS.

## Figures and Tables

**Table 1:**
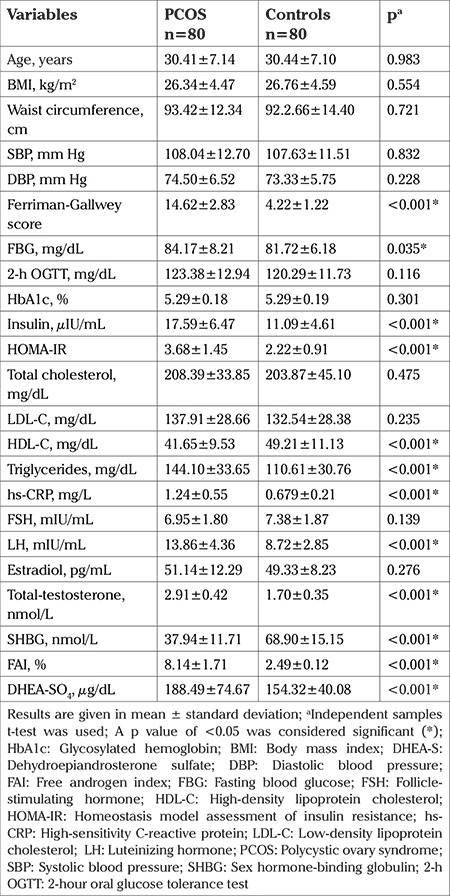
Comparison of the demographic and laboratory characteristics of the study participants

**Table 2:**
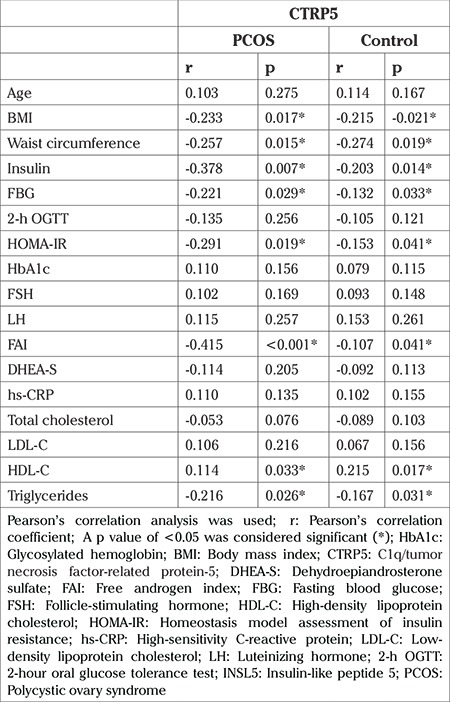
Correlation coefficient between CTRP5 levels and clinical parameters

**Table 3:**
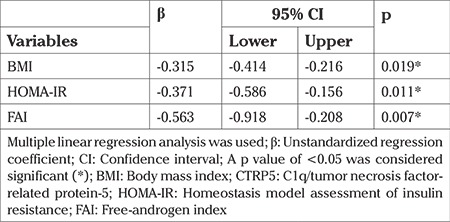
Evaluation of BMI, HOMA-IR and FAI effects on circulating CTRP5 levels in all study population using the multiple linear regression analysis (R2=0.458)
